# Acceptability of self-completion versus face-to-face use of a vertebral fragility fracture clinical decision tool for use in older people with back pain in the UK

**DOI:** 10.1007/s11657-025-01586-5

**Published:** 2025-07-23

**Authors:** Tanzeela Y. Khalid, Wendy Wilmott, Clare Shere, Tim J. Peters, Sarah Drew, Zoe Paskins, Emma M. Clark

**Affiliations:** 1 Bristol Medical School, University of Bristol, Bristol, UK; 2 Cossham Hospital, North Bristol NHS Trust, Bristol, UK; 3 Bristol Dental School, University of Bristol, Bristol, UK; 4 School of Medicine, Keele University, Staffordshire, UK

**Keywords:** Vertebral fractures, Back pain, Osteoporosis, Vfrac

## Abstract

**Summary:**

This study tested the agreement between self-completion and face-to-face completion of a vertebral fracture clinical decision tool called Vfrac in order to make an evidence-based recommendation of how Vfrac should be used for future research or clinical applications. Findings confirmed that it is necessary to take the physical measurements face-to-face.

**Background:**

Around 12% of older adults have vertebral fragility fractures but fewer than one-third are diagnosed. Vfrac is a vertebral fracture screening tool developed to help clinicians identify which patients are at a high risk of having a vertebral fracture, so they can be referred for a spinal radiograph. The aim of this work was to assess the agreement between self-completion and face-to-face use of Vfrac and determine patient preference for use.

**Methods:**

Adults aged > 65 years who had experienced back pain in the last 4 months were invited to self-complete Vfrac and have Vfrac completed face-to-face with a healthcare professional. Agreement between low risk or high risk Vfrac scores from self-completion and face-to-face assessment was represented by Cohen’s kappa; agreement in scores was also assessed between fully face-to-face and hybrid completion of Vfrac where only physical measurements are taken face-to-face and the rest self-completed. Data on satisfaction, ease of use and preference for use was also collected.

**Results:**

Data from 76 participants including 58 men and 18 women who both self-completed Vfrac and had Vfrac completed face-to-face was used to compare agreement in Vfrac scores. The mean age of participants was 76.4 years (range 65–92). There was moderate agreement in Vfrac scores (kappa 0.53; 95% confidence interval 0.31–0.75) between self-completed and face-to-face completed Vfrac with varied scores for 11 participants out of 76 (14.5%).There was only slight agreement (kappa < 0.2) for each of the three physical measurements between self-completed and face-to-face completed Vfrac. A moderate level of agreement (kappa 0.51) was also observed between fully face-to-face and hybrid completion of Vfrac. Thirty-seven percent of participants had no strong preference for how Vfrac should be completed, 33% preferred self-completion, and 30% preferred face-to-face completion.

**Conclusions:**

This study has resulted in the recommendation that future use of this tool should include completion of the physical measurements by a healthcare professional face-to-face, combined with the option of patients either self-completing the questions at home before their appointment or face-to-face at the time of the physical measurements, depending on individual preference.

**Trial registration:**

ISRCTN12150779.

## Introduction

The back is the most common site of chronic pain in the adult population living in England who have no long-lasting illness [1]. The majority of patients with insidious onset or low trauma back pain are seen by a family doctor or general practitioner (GP) in the United Kingdom (UK). National guidelines recommend a ‘wait and see approach’ for most cases of non-specific low back pain, along with self-help measures before deciding on the need for further investigation or treatment [2]. One systematic review of randomised trials found that ‘usual care’ for back pain was often highly variable in UK primary care [3].

Vertebral fragility fractures (VFFs) are present in approximately 12% of older people [4–7]. Only 25% of VFFs result from falls or trauma-related incidences, with the majority caused by daily activities such as bending forwards, climbing stairs or lifting objects [8]. Therefore, following the occurrence of VFFs, most older patients will present with back pain to their GP who they know will have a record of their past medical history. However, the majority of cases remain undiagnosed because there is a high prevalence of all-cause back pain in older people [9] and a lack of understanding about which clinical features should be used to trigger referral for a diagnostic spinal radiograph [10]. In the UK, National Institute for Health and Care Excellence guidelines advise against spinal imaging for non-specific low back pain [11] so most patients are not referred for a spinal radiograph. This is concerning because individuals who have VFFs are at high risk of further fragility fractures and if unmanaged they are likely to experience a significantly reduced health-related quality of life [12, 13]. Early diagnosis can help initiate bone-protecting treatments that reduce the risk of further fractures by 30–50% [14, 15]. To address this care gap in diagnosis, we have developed a clinical decision tool called ‘Vfrac’ to assist primary care healthcare professionals in determining if an older person with back pain requires a spinal radiograph. Vfrac consists of a series of questions and some simple physical measurements that calculate their risk of having a VFF. Vfrac has been developed using the Medical Research Council (MRC) framework for the development and evaluation of complex interventions [16] and identifies 93% of those with more than one VFF and two-thirds of those with one [17].

The delivery of primary care services has changed following the COVID-19 pandemic with many consultations now taking place remotely over the phone or by video. It is therefore prudent to assess if Vfrac can be self-completed at home to the same accuracy obtained when completed by a healthcare professional face-to-face. In particular, there is a concern that people with VFFs may find it difficult to complete one of the physical measurements (wall-to-tragus distance) due to difficulty raising their arms above head height [18]. In addition, the acceptability of completing the tool at home or face-to-face with a healthcare professional needs to be understood. Therefore, this study aims to assess: (1) the agreement between self-completion and face-to-face use of Vfrac in older men and women; (2) satisfaction and ease of use in self-completing Vfrac, acceptability of face-to-face and self-completion, and which method is preferred by patients, and (3) which completion method should be recommended for Vfrac for future research studies and/or clinical application.

## Methods

### Aim

To assess agreement of self-completion versus face-to-face use of a vertebral fracture clinical decision tool in older people with back pain (Vfrac); to assess satisfaction and ease of use of the Vfrac tool; and to make a final recommendation on how Vfrac should be used.

### Design

Testing of agreement, satisfaction, and ease of use of self-completed Vfrac compared with face-to-face assessment using a method-comparison study design with incorporated assessment of acceptability using a validated framework.

### Setting

Hospital clinic, general practice, and home.

### Regulatory approvals and registration

For pragmatic reasons, the recruitment of men and women for this research took place across two separate studies. The pragmatic reasons included the gain of added value for studies where the Vfrac clinical tool was already being used and the lack of funding for additional research in this area. Ethical approval for the study in men was obtained from the London-Bloomsbury Research Ethics Committee (REC reference 22/PR/0378). Ethical approval for the study in women was obtained from Yorkshire & The Humber – Bradford Leeds Research Ethics Committee (REC reference 22/YH/0135). The combined work to test self-completion of Vfrac with face-to-face use of Vfrac in both men and women was registered on the International Standard Randomised Controlled Trial Number registry (ISRCTN12150779) on 10th of January 2022.

### Patient and public involvement

This work was conducted in collaboration with a patient and public involvement group, called the Patient Experience Partnership in Research (PEP-R) group. Members of this group are patients who have had, or are having, treatment for musculoskeletal health conditions, including vertebral fragility fractures. The PEP-R group has worked closely with the research team on study design, delivery, interpretation, feedback and dissemination. This included revisions to the patient-facing materials such as improvements to the instructions and illustrations on how to self-complete the wall-to-tragus measurement, patient information sheet wording, study invitation letter wording, and drafting the lay summary of findings for study participants.

### The Vfrac clinical decision tool

**Fig. 1:**
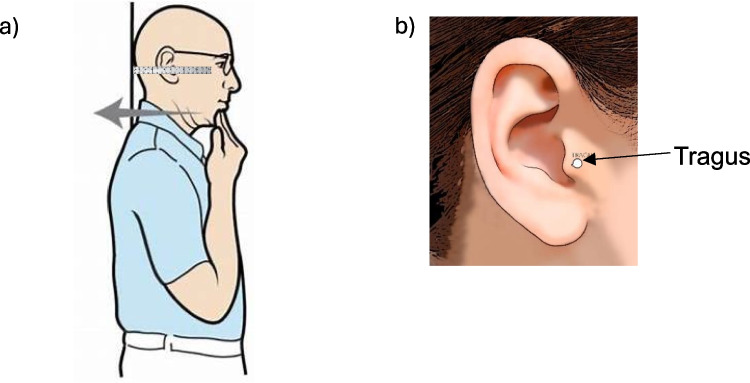
**a** Measurement of wall-to-tragus distance requires the individual to stand up straight against a wall with the buttocks touching the wall and the head positioned as far back against the wall as possible whilst tucking in the chin as much as possible. In this position, the centre of the ear and lower border of the eye socket should be level. Measure the distance between the wall and the tragus as illustrated using either a special ruler (without dead-space) or placing a piece of card against the wall and marking the position of the tragus and then measuring the distance to the marking with a ruler. **b** Measurement should be taken to the tip of the triangular part (the tragus)

As previously published [17], the Vfrac clinical decision tool consists of 12 questions and three physical measurements: height, weight and wall-to-tragus distance (Fig. 1). It is intended to help healthcare professionals decide if an older person consulting in primary care with back pain should have a spinal radiograph because they are at high risk of having a VFF. It takes 5 min to perform, and the output is a binary outcome of ‘high risk – spinal X-ray is recommended’ or ‘low risk, spinal X-ray is not recommended’ based on a pre-specified threshold. Previous published literature has reported on a method whereby patients can measure their own wall-to-tragus distance [19, 20]. These instructions were taken to a patient and public involvement meeting where seven people (including both men and women) who have had, or are having, treatment for musculoskeletal health conditions sense-checked and modified the instructions to include clear pictorial guides on how to self-measure wall-to-tragus distance. Pilot testing of these instructions was undertaken before posting them to study participants.

### Recruitment

**Fig. 2:**
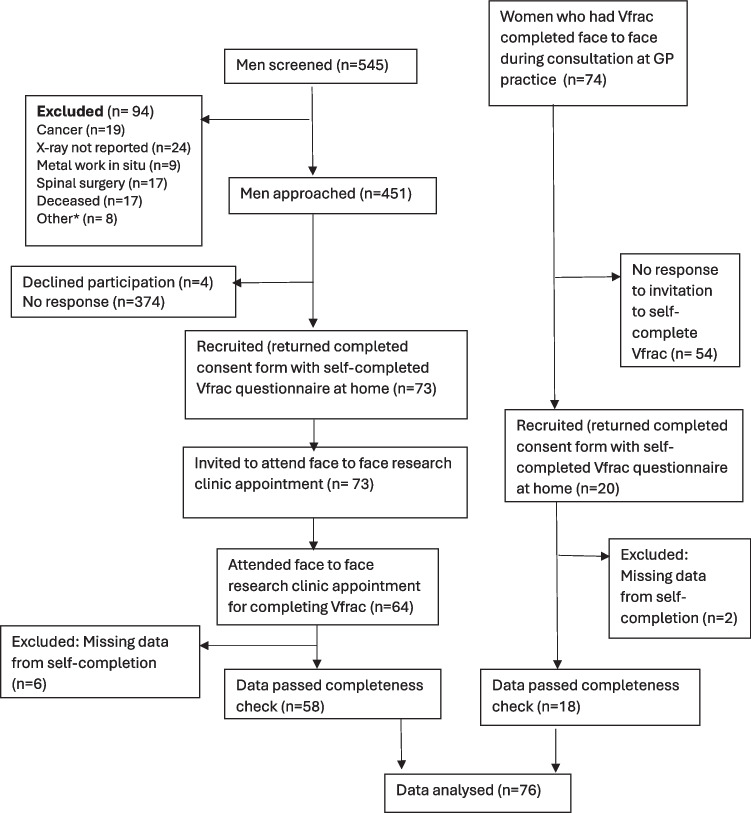
Recruitment of participants to the remote versus face-to-face testing of Vfrac. Men who had a spinal radiograph in Bristol were screened and invited to self-complete Vfrac at home, and those that did were invited to attend a clinic appointment to complete Vfrac face-to-face. Women who had Vfrac completed face-to-face during a consultation for back pain at their GP practice (as part of a separate feasibility study) were asked to self-complete Vfrac at home. Asterisk (*) denotes other exclusions (*n* = 8) include reasons such as: severe ankylosing spondylosis (*n* = 3); seriously ill (*n* = 1); mixed sclerotic and lytic appearance on radiology (*n* = 1); traumatic vertebral fragility fracture (*n* = 1); acute burst fracture (*n* = 1); not available due to imprisonment (*n* = 1)

Inclusion criteria were patients aged 65 and above, both male and female, who had an episode of back pain in the last 4 months for which they were seeking care and/or undergoing further investigations. Men aged over 65 who had a spinal radiograph in Bristol, UK, performed using the NHS standard operating procedure were identified from the digital Picture Archiving and Communication System (PACS) between August 2022 and June 2023. All men identified from PACS were first invited to self-complete Vfrac; there was no purposeful selection based on the presence or absence of vertebral fragility fractures to avoid sampling bias. Men who had a spinal malignancy or metal work in situ mentioned in their radiology report were excluded as were those who lacked the capacity to provide informed consent or were unwilling to provide informed consent (Fig. 2). Men who returned a self-completed consent form along with a paper version of the Vfrac clinical decision tool were recruited (*n* = 73) (Fig. 2). All male participants were then invited to attend a research clinic appointment where a research nurse trained to the level of a primary care practice nurse completed the Vfrac clinical tool again face-to-face, without knowing the results of the paper version to avoid confirmation bias. Sixty-four men attended this appointment.

Staff at three general practices within the NHS Bristol, North Somerset and South Gloucestershire Integrated Care Board were given access to the online Vfrac tool and were trained on the use of Vfrac as part of a separate feasibility study (ISRCTN registration number 18000119). Staff at these three GP sites were instructed to use Vfrac on all women aged 65 or over who presented with back pain between May 2023 and 2024. In total, Vfrac was used on 74 older women during their face-to-face clinical consultation where physical measurements were taken within the consultation and patient responses to all other questions were entered into the online Vfrac tool by the healthcare professional to determine the patients risk of having a current VFF. All 74 of these women were invited to take part in the present study via a postal pack that contained a study invitation letter, consent form, and paper copy of the Vfrac tool to self-complete at home. Of the 74 women invited, only 20 were recruited following the receipt of their self-completed consent form with the paper version of the Vfrac clinical decision tool (Fig. 2).

### Data collection

Self-completion data collection was via a paper copy of the published Vfrac tool [17] sent via post. To ascertain satisfaction, ease of use, and preference of completing Vfrac at home in relation to completing it face-to-face with a nurse, supplementary questions based on a framework on Quality in Healthcare to cover process, interpersonal and technical attributes [21] were included. In addition, relevant questions to assess satisfaction with telemedicine were also taken from Mekhijan et al. [22].

The face-to-face Vfrac clinical decision tool contains the same 12 questions and three physical measurements as the paper version [17]. Vfrac was completed at the research clinic for male participants, and the research nurse also verbally asked participants the following: similar questions on satisfaction with process, interpersonal and technical attributes as in the written questionnaire [21, 22]; whether they would recommend home completion, face-to-face completion by a nurse or either to friends and family; and any further comments. Female participants had the face-to-face Vfrac clinical decision tool completed as part of routine clinical care and completed supplementary questions on satisfaction on the paper form that was sent to them to complete at home.

Participants who were unable to self-complete the physical measurements (with or without assistance) were excluded from the analysis (*n* = 8). Data from face-to-face completion of Vfrac in men and self-completed Vfrac was entered into a Microsoft Access databases (Access 2016) to calculate the Vfrac output (high-risk or low-risk result) for each participant. Face-to-face Vfrac outputs for women were generated instantly using an online version of the Vfrac clinical decision tool during their consultation, and data entered on this online tool was available to the research team as a downloadable CSV file. Data from the Microsoft Access database and the online Vfrac tool was exported into Stata/MP 18.0 for data analysis.

### Sample size

The sample size was calculated as 60 based on the following assumptions: a prevalence of VFFs between 12 and 20% based on data from the European Vertebral Osteoporosis Study and the Global Burden Disease Fracture Collaborators [6, 7], a margin of error of 5%, and estimates of Cohen’s kappa in the range 0.8–0.6 (substantial agreement).

Assuming approximately 30% of the Vfrac outputs would be classified as high risk (similar to women), for a sample size of 60, the margins of error from 95% confidence intervals (CIs) around estimates of kappa in the range 0.8–0.6 (substantial agreement) would be from 0.16 to 0.22.

### Data analysis

#### Aim 1

Face-to-face completion of Vfrac was considered the gold standard. Outputs of the self-completion Vfrac (high risk versus low risk) were compared with the face-to-face Vfrac (high risk versus low risk), and agreement assessed using Cohen’s kappa. Standard classifications of Cohen’s kappa were used with a score of 0.6 or higher indicating substantial agreement. A Bland–Altman plot was used to portray the agreement between the numeric face-to-face and self-completed Vfrac scores.

A secondary analysis was undertaken to assess agreement in each of the individual question responses and physical measurements between face-to-face and self-completion of Vfrac. A paired *t*-test was used to compare the means of the face-to-face and self-completed physical measurements, and McNemar’s test was used to check for consistency of responses in the face-to-face and self-completed binary variables of the Vfrac tool.

Based on an a priori concern that patients may find it difficult to accurately undertake the three physical measurements, a third analysis was undertaken to assess if a hybrid model of self-completed questions and face-to-face completed physical measurements improves agreement with the gold standard face-to-face completion, whilst reducing the time required for a face-to-face appointment in primary care. Determining the most appropriate method of Vfrac completion is important as this will influence the MHRA approval of who Vfrac can be used on in clinical practice.

#### Aim 2

Data from the self-completed and face-to-face questionnaire on satisfaction were analysed and summarised under three themes based on the previously described frameworks [21, 22]: (1) ease of use, (2) interpersonal satisfaction, and (3) satisfaction with technical attributes. Preferences for self-completion, face-to-face, or no preference are presented as simple proportions.

#### Aim 3

A recommendation on whether the Vfrac clinical decision tool should be completed face-to-face by a healthcare professional, self-completed, or a hybrid method was based on the following: (1) the size of agreement – specifically, if Cohen’s kappa is < 0.6 (less than substantial agreement) then self-completion or hybrid methods would not be recommended and (2) patient satisfaction and ease of use of the self-completion questionnaire and written instructions.

## Results

### Study population

In total, 545 men were screened and 451 were invited to self-complete Vfrac at home. Of the 451 invited, 73 (16.2%) returned a self-completed Vfrac questionnaire (Fig. 2). All 73 men who had returned a self-completed questionnaire were invited to attend a research clinic appointment to have Vfrac completed face-to-face and in total 64 men attended. Data on measurements were missing from 6 of the self-completed questionnaires from men and so these men were excluded from the analysis (Fig. 2). Data missing included no wall-to-tragus measurement (*n* = 6), no height measurement (*n* = 2) and no weight measurement (*n* = 1). Complete data from self-completion and face-to-face completion of Vfrac was available for 58 men in total (Fig. 2).

Vfrac was used on 74 older women during a face-to-face consultation for back pain at their GP practice between March 2023 and May 2024. All these 74 women were then invited to self-complete Vfrac at home and 20 (27.0%) responded by sending back their self-completed Vfrac (Fig. 2). The wall-to-tragus measurement was missing for two women who self-completed Vfrac at home, and so they were excluded from the analysis. Data from 18 women was combined with data from 58 men to compare outputs of the self-completed Vfrac with the face-to-face Vfrac (Fig. 2).

Mean age of the combined study population was 76.4 years (range 65 to 92), and 76.3% were male. Sixty-eight of the participants in this study had a spinal X-ray (AP and lateral thoracic and lumbar views) and fifteen of these were found to have a single VFF. Self-completion of Vfrac identified 60% (9/15) of those with a single VFF similar to previous work [17] (Table 1).

**Table 1:** Cross-tabulation of spinal X-ray results with Vfrac high-risk scores following self-completion and face-to-face completion of the Vfrac clinical decision tool

Vertebral fracture diagnosis	High risk result from self-completion of Vfrac (male: female)	High risk result from face-to-face completion of Vfrac (male: female)	Total
No VFF identified on X-ray* (*n* = 61)	8 (4:4)	5(0:5)	13
Presence of VFF on spinal X-ray* (*n* = 15)	9 (6:3)	7 (4:3)	16
Total	17	12	29

^*^Spinal X-rays were carried out in 89.5% (*n* = 68) of all patients in this study (*n* = 76). The 47 patients who returned a low risk Vfrac score are not included in this table

### Agreement between face-to-face and self-completion

**Fig. 3:**
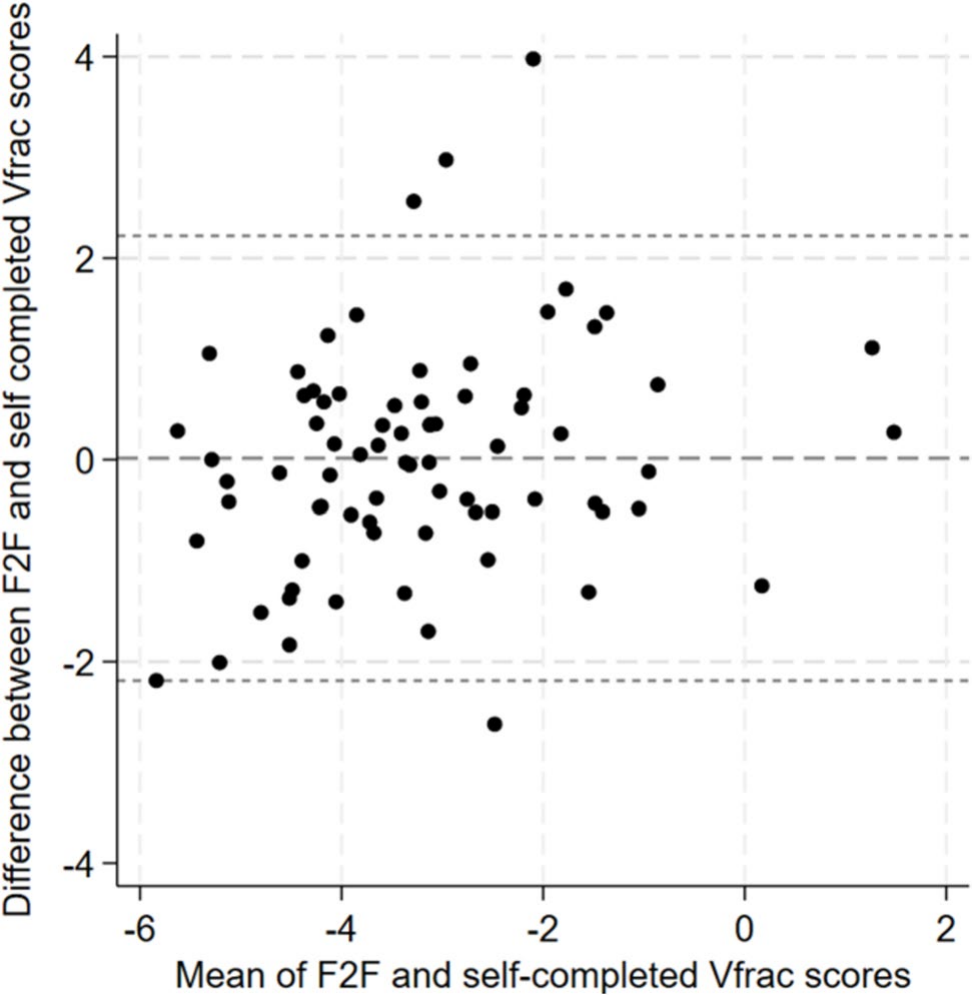
Bland–Altman plot of agreement in Vfrac scores between face-to-face (F2F) completion with a healthcare professional and self-completion at home. The middle-dashed line represents the mean difference between the two Vfrac scores, and the upper and lower dotted lines represent the 95% limits of agreement between the two methods for Vfrac completion

A Bland–Altman plot of agreement between self-completed and face-to-face completed Vfrac scores revealed no consistent bias, and likewise no apparent relationship between mean Vfrac score and the effect of the method of Vfrac completion (Fig. 3). A moderate level of agreement was found in the Vfrac outcome categorisation, with a kappa of 0.53 (95% CI 0.31 to 0.75) between self-completed and face-to-face completed Vfrac. Self-completed Vfrac identified 17 participants (22.4%) as ‘high risk of prevalent VFF’ whereas face-to-face completed Vfrac identified 12 (15.8%); see Table 2. Vfrac outcomes differed for 11 participants out of 76 (14.5%) between self-completion and face-to-face completion (Table 2).

**Table 2:** Vfrac outcomes of men and women from self-completion and face-to-face completion with a healthcare professional

	**Self-completed Vfrac**		
**Face-to-face completed Vfrac**	Low risk	High risk	Total
Low risk	56	8	64 (84.2%)
High risk	3	9	12 (15.8%)
Total	59 (77.6%)	17 (22.4%)	76 (100%)

There was only slight agreement (kappa < 0.2) for each of the three physical measurements between self-completed Vfrac and face-to-face completed Vfrac by a healthcare professional (Table 3). Responses to questions on whether sitting in straight-backed or soft chairs changed the back pain, or whether the pain can be described as like a toothache also showed slight agreement, with kappa scores ranging between 0.14 and 0.20. There was fair agreement in responses to whether or not reclining increases back pain (kappa 0.22) and whether the pain can be described as sharp (kappa 0.37). There was moderate agreement in responses to experiencing episodes of lower and upper back pain in the last 4 months, and pain that builds to a peak when standing working in the kitchen (kappa between 0.43 and 0.60). There was substantial agreement in age, taking of steroid tablets for greater than 3 months, fractures after age 50, and whether walking increases back pain, with kappa between 0.63 and 0.77.

**Table 3:** Interrater variability in the Vfrac tool variables for determination of vertebral fragility fracture risk level

Question on Vfrac tool	Self-completion (SC)	Face-to-face completion (F2F)	P value for difference	Agreement SC versus F2F	Cohen’s kappa	95% CI for Cohen’s kappa
Weight (kg)	79.0*	80.6*	0.0015	2.63%	0.025	0.015, 0.034
Wall to tragus distance (cm)	12.9†	17.0†	< 0.0001	3.95%	0.022	− 0.008, 0.052
Height loss since aged 25 (cm)	4.2*	3.4*	0.0097*	15.79%	0.106	0.060, 0.152
Age (years)	76.2*	76.5*	0.0090	64.47%	**0.630**	0.585, 0.675
Taken steroid for > 3 months	14.5%	13.2%	0.6547	93.4%	**0.724**	0.499, 0.948
Fracture after aged 50	36.8%	31.6%	0.1573	89.5%	**0.767**	0.544, 0.990
Episode of upper back pain in last 4 months	15.8%	15.8%	1.0000	89.5%	**0.604**	0.379, 0.829
Episode of low back pain in last 4 months	68.4%	73.7%	0.3458	76.3%	**0.426**	0.203, 0.649
Describes pain as sharp	48.7%	46.1%	0.6831	68.4%	0.367	0.143, 0.592
Describes pain as like toothache	72.4%	84.2%	0.0495	72.4%	0.204	− 0.008, 0.415
Pain builds to a peak when standing working in the kitchen	55.3%	60.5%	0.3173	79.0%	**0.569**	0.346, 0.793
Walking increases back pain	59.2%	55.3%	0.3657	85.5%	**0.705**	0.481, 0.929
Reclining increases back pain	39.5%	43.4%	0.5775	61.8%	0.215	− 0.009, 0.439
Sitting in straight backed chair changes back pain	63.2%	57.9%	0.4652	60.5%	0.176	− 0.047, 0.400
Sitting in soft chair changes back pain	68.4%	56.6%	0.1060	59.2%	0.143	− 0.075, 0.360

^*^Mean; †median; *p*-values for binary variables are derived from McNemar’s test. Kappa scores in bold reflect at least moderate agreement; *CI* confidence interval

### Replacement of self-completed home measurements with face-to-face clinic measurements

The three self-completed physical measurements (height, weight and wall-to-tragus distance) were replaced with face-to-face measurements to see if a hybrid method of Vfrac completion improved agreement with face-to-face completion as this is considered the gold standard. Completing Vfrac with a hybrid method, still resulted in a difference of 11 Vfrac scores compared with face-to-face completion (Table 4), so there remained only a moderate level of agreement with a kappa of 0.51 (95% CI 0.29 to 0.73). Answers to the other Vfrac questions also differed between self-completion at home and face-to-face completion with a healthcare professional. For instance, there was less than moderate agreement (kappa ≤ 0.40) in responses to questions that asked whether the pain can be described as sharp, as like a toothache, or whether back pain changed as a result of reclining or sitting in soft or straight-backed chairs (Table 3).

**Table 4:** Vfrac outcomes when completed fully face-to-face and using a hybrid method of completion

	**Hybrid completion of Vfrac**		
**Face-to-face completed Vfrac**	Low risk	High risk	Total
Low risk	57	7	64 (84.2%)
High risk	4	8	12 (15.8%)
Total	61 (80.3%)	15 (19.7%)	76 (100%)
Cohen’s kappa (95% CI)	0.51 (0.29 to 0.73)		

Hybrid method of completing Vfrac involved taking physical measurements face-to-face and self-completing the remaining questions

### Preference for Vfrac completion, satisfaction and ease of use

Participants had no strong preference for how to complete Vfrac: 25 (32.9%) preferred self-completion, 23 (30.3%) preferred face-to-face and 28 (36.8%) did not have a preference.

Free-text fields on ease of process, interpersonal satisfaction, and satisfaction with the technical attributes captured more detailed information on participants’ experiences of using Vfrac.

#### Ease around process

Most participants (89.5%) were satisfied with the written information provided for taking measurements and felt that the environment in which the questionnaire is completed was not important (Table 5). One participant commented “… very happy with the process. Very important that the process is completed by a qualified person as feel it would be more accurate”.

**Table 5:** Summary statistics on satisfaction with the process, interpersonal and technical attributes of self-completing Vfrac

	Yes *n* (%)	No *n* (%)	Missing
**Satisfaction/ease around process**			
1. Were you satisfied with written information provided for measurements?	68 (89.5%)	8 (10.5%)	0 (0%)
2. Do you think the environment where the questionnaire is completed is important?	21 (27.6%)	44 (57.9%)	11 (14.5%)
**Satisfaction around interpersonal attributes**			
1. Were you comfortable completing all the questions at home?	69 (90.8%)	7 (9.2%)	0 (0%)
2. Would you feel more comfortable completing the whole questionnaire face-to-face with a nurse?	33 (43.4%)	43 (56.6%)	0 (0%)
3. Is the value of personal relationships important to you when deciding how to complete this questionnaire?	43 (56.6%)	21 (27.6%)	12 (15.8%)
4. Is the value of face-to-face communication important to you when deciding how to complete this questionnaire?	66 (86.8%)	4 (5.3%)	6 (7.9%)
**Satisfaction around technical attributes**			
1. Were you able to do the three measurements (height, weight and wall-to-tragus distance)?	61 (80.3%)	15 (19.7%)	0 (0%)
**Recommendations for completion of questionnaire by friends and family**	Self-complete at home	Complete face-to-face with a nurse	Either
1. I would recommend the following to my friends and family	19 (25.0%)	25 (32.9%)	32 (42.1%)

### Satisfaction around interpersonal attributes

Most (90.8%) were comfortable completing all the questions at home (Table 5), feeling that completing it face-to-face with a nurse would not make it more comfortable. However, most participants also agreed that the value of personal relationships and face-to-face communication were important factors when deciding how to complete this questionnaire. Participants who indicated their preference to complete Vfrac face-to-face with a nurse included reasons such as more certainty of measurements being accurate, difficulty doing measurements on their own (especially the wall-to-tragus distance), being able to provide more in-depth information about the pain they experience, having the opportunity to ask questions and discuss responses to some questions, especially where there is not a clear ‘Yes’ or ‘No’ response. One participant commented “more value to all parties in meeting face-to-face … participants with back pain feel it’s more beneficial to talk face-to-face”.

### Satisfaction around technical attributes

Around 80% of participants reported, they were able to do the three measurements (Table 5). Participants who reported they were unable to do the three measurements had still provided sensible measurements, with most indicating that they had not be able to self-complete Vfrac without assistance from a family member to help take measurements. One of the participants commented “… all seemed readily doable. Unaided person may find measurements difficult, is this taken into account?”.

Nine participants found it difficult and/or painful to stand in the required position for height and wall-to-tragus measurements. One participant commented “… as an older woman my balance is less good than it was, I am not unusual. I think assistance should be recommended in older participants. Information was clearly explained but could not be done without help. Also skirting boards mean you can’t put your heels up to the wall”. Another male participant commented “… found it difficult to measure the distance for tragus as I was unable to touch the wall with my head”.

### Recommendation for completion of Vfrac

Recommendations to friends and family on how to complete Vfrac varied and supported the view that both self-completion and face-to-face completion were good options. Other factors that participants deemed to be important when making a decision on how to complete Vfrac included taking accurate measurements (73.7% agreed this was important), ease and comfort (57.9%), and difficulty with self-completion (51.3%). Travel to a clinic was considered less important (27.6%).

Questions on satisfaction and ease of use revealed that self-completion of Vfrac at home is suitable for some participants, but difficult for some with regard to completing the physical measurements and almost impossible for some without assistance in this respect.

## Discussion

This small study identified a moderate level of agreement between self-completion and face-to-face completion of Vfrac. Participants generally found the tool acceptable to self-complete at home or face-to-face with a healthcare professional. Hybrid completion of Vfrac, whereby physical measurements taken face-to-face are combined with self-completed responses to other questions, also showed a moderate level of agreement to face-to-face Vfrac scores. There was only slight agreement in the three physical measurements when comparing self-completion with face-to-face completion.

We had an a priori concern that the physical measurements may be difficult, particularly for the wall-to-tragus measurement, since people with vertebral fractures have difficulty raising their arms above head height [23]. Around 20% of participants experienced difficulties with taking their measurements at home mentioning the requirement for assistance, and around 10% were unable to complete their wall-to-tragus measurement and had to be excluded from this study. It can be assumed that some of the non-responders were unable to complete their physical measurements. Due to ethical and time constraints, we were unable to quantify what proportion of the non-responders were unable to complete their measurements, and whether this was the sole reason for not responding to the study invitation. Around 7% of participants reported height gain instead of height loss since the age of 25, and measurements for weight were generally higher when taken face-to-face compared with self-completion.

Taking into account the difficulty described by some participants with self-completion of their physical measurements, and the only slight agreement in physical measurements between face-to-face and self-completion (kappa < 0.2), we recommend that all three physical measurements are taken face-to-face by a healthcare professional, with the option of either completing the remaining questions with the same healthcare professional face-to-face or independently based on individual preference. In support of this recommendation, we note that the kappa statistics in Tables 2 and 4 are virtually identical, and so there would be no loss of underlying agreement using the hybrid approach whilst at the same time increasing the acceptability of the approach and potentially the accuracy and reliability of at least some of the scores.

Some participants in this study had a time delay of around 3 months between self-completion and face-to-face completion of Vfrac, and so it is possible that the level of agreement may have been attenuated by changes in the participant’s clinical status with regard to experiences of pain. Differences in physical measurements may also be expected when you have trained/untrained personnel taking measurements plus different measuring equipment. Self-reported measurements of height and weight frequently show discrepancies compared with face-to-face measurements [24, 25]. A systematic review of large epidemiological studies that required participants to self-report measures of height and weight found a trend where women underreported their weight, whilst men overreported their height [26]. We thus recommend that the physical measurements for Vfrac completion are obtained face-to-face by a healthcare professional.

Strengths of this study are the age range of participants (up to age 92) and the important recommendation outcome that should influence future use of the Vfrac tool and potential licensing approvals. Limitations include the lack of data on ethnicity, the low proportion of women particularly as women report more chronic pain than men [1], and different recruitment methods for older men and women. This study recruited men who has already been referred for a spinal X-ray meaning their back pain was significant enough to warrant further investigation and thus it is important to note that this might not be representative of the older male patients that present to primary care.

For pragmatic reasons, the recruitment of men and women for this research took place across two separate studies where self-completion of Vfrac and face-to-face completion of Vfrac took place in the opposite order. Women already had Vfrac completed face-to-face prior to self-completion at home, so they may have been more informed than the men.

The prevalence of VFF in this dataset was 15/76 (19.7%), which is in line with that reported for women in this age group [6, 7]. However, this study was not powered to test the accuracy of Vfrac for the prediction of those at high risk of VFF, so the diagnostic accuracy of the self-completion, face-to-face, and hybrid methods of completion were not calculated. Data on the ethnicity of participants was not collected, and it is likely that the recruited population is not fully representative of the local population. It is imperative to address the systematic exclusion of particular groups from research which limits the generalizability of research findings and perpetuates health inequalities. Thus, Vfrac has now been translated into Urdu, Punjabi and Bengali using a cross-cultural adaptation approach [27]. Further research involving the Vfrac clinical decision tool will consider research equity at the outset.

The low proportion of women participants may also have an implication for generalisability, but we think the risk of this is low. The underlying biology of osteoporosis and VFFs is similar in men and women [28], and the spinal shape changes are likely to be similar, suggesting difficulties with the physical measurements are likely to be similar. There is also evidence for greater variability between self-completion and face-to-face completion of questionnaires with men [29], so the addition of more women to our study population would have likely improved the level of agreement. This is reassuring for our recommendation of a hybrid or face-to-face method of Vfrac completion. More work is required to assess how well Vfrac works in older men compared to older women given that the numbers recruited in this study were not large enough to make that comparison.

## Conclusions

This study of self-completion versus face-to-face assessment of the Vfrac clinical decision tool has resulted in the recommendation that future use of this tool should include completion of the physical measurements by a healthcare professional, combined with the option of patients self-completing the questions at home prior to their appointment or at the time of the physical measurements, depending on individual preference. This recommendation is supported by the finding that there would be no loss of underlying agreement using the hybrid approach whilst at the same time increasing the acceptability for some patients. This is important information for the future definitive evaluation of the clinical and cost-effectiveness of Vfrac during which MHRA approval of this as a Class I device will be obtained. The method of Vfrac data collection utilising a hybrid approach as recommended by this research study should therefore influence the MHRA approval of who Vfrac can be used on in clinical practice.

## Data Availability

The anonymised dataset supporting the conclusions of this article will be available in the University of Bristol Research Data Repository within 6 months following publication. Data will be restricted and only made available to bona fide researchers for ethically approved research projects after a data access agreement has been signed by an institutional signatory.

## References

[1] Public Health England (2017) Chronic pain in adults 2017 - Health Survey for England. In: pp. 23. https://assets.publishing.service.gov.uk/media/5fc8c6b78fa8f547585ed7f3/Chronic_Pain_Report.pdf.

[2] National Institute for Health and Care Excellence (2020) Low back pain and sciatica in over 16s: assessment and management (NG59). In: Guidance pp 1–23. https://www.nice.org.uk/guidance/ng59/resources/low-back-pain-and-sciatica-in-over-16s-assessment-and-management-pdf-1837521693637 33090750

[3] Somerville S, Hay E, Lewis M, Barber J, van der Windt D, Hill J, Sowden G (2008) Content and outcome of usual primary care for back pain: a systematic review. Br J Gen Pract 58(556):790–797 19000402 10.3399/bjgp08X319909 PMC2573978

[4] Clark EM, Hutchinson AP, McCloskey EV, Stone MD, Martin JC, Bhalla AK, Tobias JH (2010) Lateral back pain identifies prevalent vertebral fractures in post-menopausal women: cross-sectional analysis of a primary care-based cohort. Rheumatology (Oxford) 49(3):505–512 20015975 10.1093/rheumatology/kep414 PMC2895162

[5] Drew S, Clark E, Al-Sari U, Moore A, Gooberman-Hill R (2020) Neglected bodily senses in women living with vertebral fracture: a focus group study. Rheumatology (Oxford) 59(2):379–385 31335949 10.1093/rheumatology/kez249

[6] O’Neill TW, Felsenberg D, Varlow J, Cooper C, Kanis JA, Silman AJ (1996) The prevalence of vertebral deformity in european men and women: the European Vertebral Osteoporosis Study. J Bone Miner Res 11(7):1010–1018 8797123 10.1002/jbmr.5650110719

[7] Global Burden Disease Fracture Collaborators (2021) Global, regional, and national burden of bone fractures in 204 countries and territories 1990–2019: a systematic analysis from the global burden of disease study 2019. Lancet Healthy Longev 2(9):e580–e592 34723233 10.1016/S2666-7568(21)00172-0 PMC8547262

[8] Holroyd C, Cooper C, Dennison E (2008) Epidemiology of osteoporosis. Best Pract Res Clin Endocrinol Metab 22:671–685. 10.1016/j.beem.2008.06.001 19028351 10.1016/j.beem.2008.06.001

[9] Wong AY, Karppinen J, Samartzis D (2017) Low back pain in older adults: risk factors, management options and future directions. Scoliosis and spinal disorders 12:14. 10.1186/s13013-017-0121-3 28435906 10.1186/s13013-017-0121-3 PMC5395891

[10] Clark EM, Cummings SR, Schousboe JT (2017) Spinal radiographs in those with back pain-when are they appropriate to diagnose vertebral fractures? Osteoporos Int 28:2293–2297. 10.1007/s00198-017-4052-x 28444431 10.1007/s00198-017-4052-x

[11] National Institute for Health and Care Excellence (2024) Back pain-low (without radiculopathy): How should I assess a person with low back pain? Clinical Knowledge Summaries [Accessed: 28/06/2025]. Available at: https://cks.nice.org.uk/topics/back-pain-low-without-radiculopathy/diagnosis/assessment/. Last revised in October 2024

[12] Johansson L, Svensson HK, Karlsson J, Olsson LE, Mellström D, Lorentzon M, Sundh D (2019Oct) Decreased physical health-related quality of life-a persisting state for older women with clinical vertebral fracture. Osteoporos Int 30(10):1961–1971. 10.1007/s00198-019-05044-0 31227884 10.1007/s00198-019-05044-0 PMC6795611

[13] Stanghelle B, Bentzen H, Giangregorio L, Pripp AH, Bergland A (2019Nov 4) Associations between health-related quality of life, physical function and pain in older women with osteoporosis and vertebral fracture. BMC Geriatr 19(1):298. 10.1186/s12877-019-1268-y 31684886 10.1186/s12877-019-1268-y PMC6829800

[14] Boonen S, Laan RF, Barton IP, Watts NB (2005) Effect of osteoporosis treatments on risk of non-vertebral fractures: review and meta-analysis of intention-to-treat studies. Osteoporos Int 16(10):1291–1298 15986101 10.1007/s00198-005-1945-x

[15] Watts NB, Josse RG, Hamdy RC, Hughes RA, Manhart MD, Barton I, Calligeros D, Felsenberg D (2003) Risedronate prevents new vertebral fractures in postmenopausal women at high risk. J Clin Endocrinol Metab 88(2):542–549 12574177 10.1210/jc.2002-020400

[16] Craig P, Dieppe P, Macintyre S, Michie S, Nazareth I, Petticrew M (2008) Developing and evaluating complex interventions: the new Medical Research Council guidance. BMJ 337:a1655 18824488 10.1136/bmj.a1655 PMC2769032

[17] Khera TK, Hunt LP, Davis S, Gooberman-Hill R, Thom H, Xu Y, Paskins Z, Peters TJ, Tobias JH, Clark EM (2022) A clinical tool to identify older women with back pain at high risk of osteoporotic vertebral fractures (Vfrac): a population-based cohort study with exploratory economic evaluation. Age Ageing 51(3):afac031. 10.1093/ageing/afac031 35284926 10.1093/ageing/afac031 PMC8918203

[18] Al-Sari UA, Tobias JH, Clark EM (2018) Self-reported everyday physical activities in older people with osteoporotic vertebral fractures: a systematic review and meta-analysis. Osteoporos Int 29:19–29. 10.1007/s00198-017-4287-6 29098348 10.1007/s00198-017-4287-6

[19] Mani S, Sharma S, Omar B, Paungmali A, Joseph L (2017) Validity and reliability of Internet-based physiotherapy assessment for musculoskeletal disorders: a systematic review. J Telemed Telecare 23(3):379–391 27036879 10.1177/1357633X16642369

[20] Truter P, Russell T, Fary R (2014) The validity of physical therapy assessment of low back pain via telerehabilitation in a clinical setting. Telemed J E Health 20(2):161–167 24283249 10.1089/tmj.2013.0088

[21] Huycke L, All AC (2000) Quality in health care and ethical principles. J Adv Nurs 32(3):562–571 11012797 10.1046/j.1365-2648.2000.01540.x

[22] Mekhjian H, Turner JW, Gailiun M, McCain TA (1999) Patient satisfaction with telemedicine in a prison environment. J Telemed Telecare 5(1):55–61 10505370 10.1258/1357633991932397

[23] Al-Sari UA, Tobias JH, Clark EM (2018) Self-reported everyday physical activities in older people with osteoporotic vertebral fractures: a systematic review and meta-analysis. Osteoporos Int 29(1):19–29 29098348 10.1007/s00198-017-4287-6

[24] Bowring AL, Peeters A, Freak-Poli R, Lim MS, Gouillou M, Hellard M (2012) Measuring the accuracy of self-reported height and weight in a community-based sample of young people. BMC Med Res Methodol 12:175 23170838 10.1186/1471-2288-12-175 PMC3561081

[25] Flegal KM, Ogden CL, Fryar C, Afful J, Klein R, Huang DT (2019) Comparisons of self-reported and measured height and weight, BMI, and obesity prevalence from national surveys: 1999–2016. Obesity (Silver Spring) 27(10):1711–1719 31544344 10.1002/oby.22591 PMC7289317

[26] Fayyaz K, Bataineh MF, Ali HI, Al-Nawaiseh AM, Al-Rifai RH, Shahbaz HM (2024) Validity of measured vs self-reported weight and height and practical considerations for enhancing reliability in clinical and epidemiological studies: a systematic review. Nutrients 16(11):1704. 10.3390/nu16111704 38892637 10.3390/nu16111704 PMC11175070

[27] Beaton DE, Bombardier C, Guillemin F, Ferraz MB (2000) Guidelines for the process of cross-cultural adaptation of self-report measures. Spine (Phila Pa 1976) 25(24):3186–3191 11124735 10.1097/00007632-200012150-00014

[28] Alswat KA (2017) Gender disparities in osteoporosis. J Clin Med Res 9(5):382–387 28392857 10.14740/jocmr2970w PMC5380170

[29] Rohde P, Lewinsohn PM, Seeley JR (1997) Comparability of telephone and face-to-face interviews in assessing axis I and II disorders. Am J Psychiatry 154(11):1593–1598 9356570 10.1176/ajp.154.11.1593

